# Supplementary material for: The impact of high-temperature treatments on maize growth parameters and soil nutrients: A comprehensive evaluation through principal component analysis
Source: PLoS One. 2024 Aug 15;19(8):e0309070. doi: 10.1371/journal.pone.0309070 (PMC11326588; doi:10.1371/journal.pone.0309070)
Supplement: Attachment — Submitted filename: Response to Reviewers.docx [file pone.0309070.s003.docx]

Dear editors and reviewers,

Thank you for your review of our paper and your valuable feedback. We have seriously considered your suggestions and have further reviewed and revised our research with a view to improving the quality and science of the paper. The following is a one-to-one reply comments and amendment instructions, please review.

Reviewer #1: The manuscript titled " Evaluating the impact of high-temperature treatments at different times on soil comprehensive quality based on principal component analysis" has been reviewed. The study is a current scientific research focus. The material and methods are standards and the outcome / results justified the methods use. However, this paper requires major revision to improve the manuscript:

1. Line number 29, suggested to write the control rather than CK

Response: The author has changed CK to control.

2. Cite the following articles to improve the manuscript with updated literature.

https://doi.org/10.1080/15226514.2024.2310001

https://doi.org/10.1016/j.apsoil.2022.104680

https://doi.org/10.1016/j.scitotenv.2023.167756

https://doi.org/10.1016/j.scitotenv.2023.169420

https://doi.org/10.1016/j.stress.2023.100345

https://doi.org/10.1016/j.jhazmat.2021.127021

Response: The author cited the latest references above, and the novelty of the article has been improved. Thank you for your advice.

3. There are numerous inappropriately written English syntax and words. All sections of the manuscript suggested to revised for English language correctness.

Response: The author has asked native English speakers to modify the language of the article, and it is believed that the readability and logic of the article language will be enhanced after the author's efforts to modify it.

4. Line 71, suggested to write proper citation in the text.

Response: The author has revised this sentence and cited the literature. “In addition, temperature and heating time also have an impact on crop growth and yield, and studies have shown that instantaneous temperature and shorter heating time can stimulate crop growth and development, thereby increasing yield [16].”

5. Introduction lacks information regarding maize crops. Suggested to add some sentences about the importance of maize crops in the introduction section.

Response: Authors have been added: “Maize (Zea mays), is one of the most vital cereal crops worldwide, serving as a staple food for millions of people and playing a crucial role in global agriculture and economies [7]. Especially in developing countries, maize is a major food source and a major component of livestock feed. As an adaptable crop, maize has a wide range of adaptability to the soil, but in the barren land, it still needs to be properly managed and adjusted to maximize its growth potential [8].”

6. It is proposed to more clearly elaborate the study hypothesis at the end of introduction section.

Response: The author has revised the purpose and hypothesis of this study at the end of the introduction: “The objectives of this study were to (1) investigate the effects of temperature treatments on soil properties over varying time scales; (2) clarify the response of maize growth indicators to temperature gradients; (3) clarify the optimal temperature gradient under high-temperature treatments. We hypothesized that (1) the

high temperature treatment can promote the comprehensive quality of soil and maize; (2) the short-term high temperature treatments would be more effective.”

7. Why authors used maize crops as test plant for the experiment?

Response: Widespread Cultivation: Maize is one of the most widely cultivated crops globally, making it readily available for experimentation and ensuring that findings can be applicable across different regions where maize is grown. And it is the main food crop in the region studied by the authors.

Experimental Convenience: Maize plants have relatively short growth cycles and are amenable to experimental manipulations, making them suitable for controlled environment studies aimed at investigating the effects of temperature on plant physiology and soil properties.

8. Results are not still properly discussed based on the latest studies.

Response: The author has fully discussed the results of the study, and rewrote the discussion part from three aspects: plant index, soil index and comprehensive evaluation result.

9. Authors are suggested to consider reinforcing the manuscript with related and useful literature published in 2023, 2024.

Response: The author has added new references.

10. After corrections and revisions, the whole manuscript should be edited and proofread.

Response: According to the reviewer's professional opinion, the author made detailed revisions to the article.

11. Authors are suggested to improve the conclusion section, it looks repetition of results.

Response: The author has revised the conclusion.

Reviewer #2: The title and short title are not matching. The authors are requested to pleases rewrite the title for better clarity.

Response: The author made changes.

Title: The combined effect of temperature on soil and maize was evaluated based on principal component analysis

Short title: High temperature impact on crops and soil

The hypothesis is very poor, it is difficult to establish the relation between the rise in soil temp and crop growth, to a certain extent rise in soil temperature may help in checking the soil pathogens but it also accelerate the losses of soil nutrients. The increases of soil temperatures cant not be a sustainable practice to manage soil pathogens. It can be understood that from large scale burning of crop residues in many parts of South east Asia, especially in India. This practices has been vividly criticized for negative impact on soil and other agro climatic conditions.

Response: The authors rewrote the purpose and hypothesis of the study so that the results could answer the hypothesis. “We hypothesized that (1) the high temperature treatment can promote the comprehensive quality of soil and maize; (2) the short-term high temperature treatments would be more effective.”

This study, the high temperature treatment is heated in the surface layer of soil, but the temperature conduction to the lower layer of soil will not produce several hundred degrees of temperature, which is obviously the soil played a buffering role. At this time, the excessive loss of nutrients will not be aggravated. On the contrary, the appropriate temperature in the soil layer stimulates the microbial activity and optimizes the root growth zone, which may promote the improvement of the overall quality of crops and soil. Of course, this is only one reason. The deeper reasons need to be explored and experimented continuously, which is one of the future research directions of the author.

In Table 2. Soil physical and chemical indicators under different temperature and time treatments, it is highly unusual to understand the effect of temperature on different soil parameters'. How soil texture is being impacted due to rise in soil temperatures that too for few second. This type of effects of rise in soil temperature are highly unusual.

Response: Because temperature treatment potentially affects many factors such as soil internal elements, microorganisms, enzyme activities, stomata and so on, changes in these factors will inevitably bring changes in soil properties and crop indicators. The author collected the rhizosphere soil after the completion of maize growth to reveal the index changes more sensitively. The results showed that the soil texture was only a small difference in soil particles and did not change.

Soil texture is a critical component of soil health, influencing factors such as water retention, nutrient availability, and root penetration. While it may seem unexpected for brief temperature fluctuations to affect soil texture, it's important to consider the potential mechanisms driving these changes. One possibility is that rapid heating could alter the arrangement of soil particles or induce temporary changes in soil structure, leading to observable differences in texture.

Additionally, it's essential to recognize the interconnected nature of soil properties and processes. Changes in temperature can influence microbial activity, organic matter decomposition, and chemical reactions within the soil, all of which may indirectly affect soil texture over time.

However, further research is needed to fully understand the mechanisms behind the observed effects and to determine the long-term implications for soil health and agricultural productivity. Conducting controlled experiments and monitoring soil properties over extended periods under varying temperature conditions could provide valuable insights into these phenomena.

The various results as shown by the authors in different tables are not acceptable mainly because of scientific validity and and very superficial explanations.

Response: The authors present the values of these indicators in a table. On this basis, a series of tests and evaluations were made using principal component analysis, and the final conclusion was obtained by the principal component statistical analysis method, which was used to determine the significant differences between different treatments. We will also take care to ensure that data and statistical analysis results are presented more clearly in future writing to improve the readability and scientific nature of the paper. Thank you.
