# Supplementary material for: The impact of high-temperature treatments on maize growth parameters and soil nutrients: A comprehensive evaluation through principal component analysis
Source: PLoS One. 2024 Aug 15;19(8):e0309070. doi: 10.1371/journal.pone.0309070 (PMC11326588; doi:10.1371/journal.pone.0309070)
Supplement: Attachment — Submitted filename: Review Comments_PONE-D-24-13037.docx [file pone.0309070.s004.docx]

**THE COMBINED EFFECT OF TEMPERATURE ON SOIL AND MAIZE WAS EVALUATED BASED ON PRINCIPAL COMPONENT ANALYSIS- PONE-D-24-13037**

This study investigated the impact of temperature-modified soil on nutrient activation and crop growth, using loess soil with low fertility and maize as the test plant. Overall, the paper was well written, with clear objectives, detailed methodology, and systematic description of the results.

**Overall Decision: Minor revision**

**TOPIC**

1. Rephrase the topic. It currently reads like the objective of the study
2. Arrange key words in alphabetical order

**ABSTRACT & KEYWORDS**

1. Lines 14-16: The problem statement is not clearly defined

**INTRODUCTION**

1. Line 79-80. *Using the principal component analysis method to evaluate soil physicochemical properties and crop growth indexes*. The sentence is hanging. Rephrase.
2. Overall, the Introduction section is well written

**MATERIALS AND METHODS**

1. Lines 89-98: What is general classification of the soil with reference to common nomenclature e.g. WRBS (FAO), USDA
2. Lines 89-98: Rationale for site selection should be further strengthened. What does the Loess Plateau represent in the broader crop production systems in China and similar farming systems in the world?
3. Lines 101-113: What informed/guided the temperature and heating periods used in the study
4. It’s not clearly stated in the Materials and Methods the time period (duration of the experiment) the experiment was done. In addition, it is important to provide information of the days to maturity of the maize variety used in this study
5. Was the soil characterized for physicochemical and biological properties before experimentation?

**RESULTS & DISCUSSION**

1. Tables 1 and 2: Were the treatments significantly different? There no statistical indices for comparison of treatment means.
2. Limitations of the study and grey areas for further research should be clearly articulated in the Discussion.

- The study was conducted under controlled (pot experiment) conditions, which does not represent the real field conditions where farmers grow maize. It therefore becomes challenging to extrapolate the findings to field conditions where both biotic and abiotic conditions vary in space and time.
- Measurements were only done over one cropping cycle. The study could have been conducted over 2 or more cropping cycles to validate the results. In the absence of these repeated measurements over 2 or more seasons, the authors could consider simulation modeling to validate their findings in space and time
- Limitations of Principal Component Analysis in the context of this study

3. I was wondering, what are the implications of the study findings regarding management of temperature regimes in soils and impacts on soil chemical and biological properties in maize-based cropping systems. For example, how can farmers realistically manipulate soil temperatures to influence soil chemical and biological properties and crop growth?
