# Supplementary material for: The impact of high-temperature treatments on maize growth parameters and soil nutrients: A comprehensive evaluation through principal component analysis
Source: PLoS One. 2024 Aug 15;19(8):e0309070. doi: 10.1371/journal.pone.0309070 (PMC11326588; doi:10.1371/journal.pone.0309070)
Supplement: Attachment — Submitted filename: Response to Reviewers.docx [file pone.0309070.s005.docx]

**Response to Reviewers**

Dear Editors and Reviewers,

The authors would like to thank the reviewers for their valuable input. We have addressed all of the comments as completely as possible. Changes are shown in red in the manuscript. Below are responses to individual comments. Overall, the experimental methods have been explained much more thoroughly, and the manuscript has been edited by a professional whose native language is English. These revisions have greatly improved the quality of the manuscript, and we hope that the manuscript now meets the high standards of PLOS One. The response to each individual comment is shown below.

PONE-D-24-13037

The combined effect of temperature on soil and maize was evaluated based on principal component analysis

PLOS ONE

Dear Dr. Han,

Thank you for submitting your manuscript to PLOS ONE. After careful consideration, we feel that it has merit but does not fully meet PLOS ONE’s publication criteria as it currently stands. Therefore, we invite you to submit a revised version of the manuscript that addresses the points raised during the review process.

Comments from the editorial office: One or more of the reviewers has recommended that you cite specific previously published works in your original submission PONE-D-24-07715 such as : 10.1080/15226514.2024.2310001; 10.1016/j.apsoil.2022.104680; 10.1016/j.scitotenv.2023.167756; 10.1016/j.scitotenv.2023.169420; 10.1016/j.stress.2023.100345; 10.1016/j.jhazmat.2021.127021 . Members of the editorial team have determined that the works referenced are not directly related to the submitted manuscript. As such, please note that it is not necessary or expected to cite the works requested by the reviewer and these may be removed from your submission if you wish.

**Response:** The author carefully revised the references based on the editor's feedback.

References 1, 5, 10, 27, and 30, which are closely related to the content of the article, were cited.

If applicable, we recommend that you deposit your laboratory protocols in protocols.io to enhance the reproducibility of your results. Protocols.io assigns your protocol its own identifier (DOI) so that it can be cited independently in the future. For instructions see: https://journals.plos.org/plosone/s/submission-guidelines#loc-laboratory-protocols. Additionally, PLOS ONE offers an option for publishing peer-reviewed Lab Protocol articles, which describe protocols hosted on protocols.io. Read more information on sharing protocols at https://plos.org/protocols?utm_medium=editorial-email&utm_source=authorletters&utm_campaign=protocols.

**Response:** In the materials and methods section, the author provided a detailed description of the experimental design, operation procedure, and detection method related to the experimental protocols in this article.

Journal requirements:

When submitting your revision, we need you to address these additional requirements.

1. Please ensure that your manuscript meets PLOS ONE's style requirements, including those for file naming. The PLOS ONE style templates can be found at

https://journals.plos.org/plosone/s/file?id=wjVg/PLOSOne_formatting_sample_main_body.pdf and https://journals.plos.org/plosone/s/file?id=ba62/PLOSOne_formatting_sample_title_authors_affiliations.pdf

**Response:** Our manuscript meets the stylistic requirements of PLOS ONE.

2. PLOS requires an ORCID iD for the corresponding author in Editorial Manager on papers submitted after December 6th, 2016. Please ensure that you have an ORCID iD and that it is validated in Editorial Manager. To do this, go to ‘Update my Information’ (in the upper left-hand corner of the main menu), and click on the Fetch/Validate link next to the ORCID field. This will take you to the ORCID site and allow you to create a new iD or authenticate a pre-existing iD in Editorial Manager. Please see the following video for instructions on linking an ORCID iD to your Editorial Manager account: https://www.youtube.com/watch?v=_xcclfuvtxQ.

**Response:** The author has provided ORCID iD: 0000-0002-1609-9848

**Reviewer #1**

Kindly check the sentences and spell check like in line number 57-58 in introduction. It has been mentioned he high-temperature treatment is a commonly used method in soil facility management. It should be soil fertility not soil facility.

**Response:** The author has revised to replace "soil facility" with "soil fertility".

Similarly in first line of introduction, it will be more appropriate to use soil fertility instead of soil infertility in line 41-42, The expansion of large-scale and intensive agriculture has worsened soil infertility due to unsustainable farming practices"

**Response:** The author has revised the sentence “The expansion of large-scale and intensive agriculture has negatively impacted soil fertility through unsustainable farming practices".

As also earlier revisions, it was asked to explain whether rising soil temperature is s sustainable method of managing the soil pathogens. what was the effect of all these temperature treatments on soil beneficial microbes, soil enzymatic activities etc.

**Response:** The last paragraph of the introduction has been rewritten by the author to answer the questions raised by the reviewers.

“Soil temperature and heating duration are key factors affecting the physicochemical characteristics of soil and crop development. **The short-term exposure to high temperatures can quickly enhance the soil conditions, proving to be a more effective approach than prolonged high temperature maintenance throughout the entire growth cycle**. The short high temperature treatment can stimulate soil microbial activity, support the proliferation and function of beneficial microorganisms, and increase the activity of enzymes, thereby accelerating the decomposition of organic matter”.

It is also not a usual impact of increase in soil temperature on root and shoot parameters.

The data to give information in support of defined objectives need better clarity. especially on The objectives of this study were to (1) investigate the effects of temperature treatments on soil properties over varying time scales; (2) clarify the response of maize growth indicators to temperature gradients; (3) clarify the optimal temperature under high-temperature treatments. We hypothesized that (1) the high temperature treatment can promote the comprehensive quality of soil and maize; (2) the short-term high temperature treatments would be more effective.

**Response:** In this study, the soil surface underwent high temperature treatment, impacting the activities of microorganisms and enzymes, consequently affecting soil properties. Subsequently, maize was planted in the cooled soil treated with high temperature to observe growth differences, representing the innovation of this research. As a result, the author adjusted the study's purpose to highlight these distinctions.

“The objectives of this study were to (1) investigate the impact of short-term high-temperature treatment on soil properties; (2) explore the difference between planting maize in soil treated with high temperatures; (3) identify the optimal combination of temperature and duration. We hypothesized that (1) the high temperature treatment can promote the comprehensive quality of soil and maize; (2) the short-term high temperature treatments would be more effective”.

**Reviewer #2**

THE COMBINED EFFECT OF TEMPERATURE ON SOIL AND MAIZE WAS EVALUATED BASED ON PRINCIPAL COMPONENT ANALYSIS- PONE-D-24-13037

This study investigated the impact of temperature-modified soil on nutrient activation and crop growth, using loess soil with low fertility and maize as the test plant. Overall, the paper was well written, with clear objectives, detailed methodology, and systematic description of the results.

Overall Decision: Minor revision

**Response:** Thank you for your comments, I will modify and improve according to your comments.

TOPIC

1. Rephrase the topic. It currently reads like the objective of the study

2. Arrange key words in alphabetical order

**Response:** 1. The author revised the title according to the opinion. Title: The impact of high-temperature treatments on maize growth parameters and soil nutrients: A comprehensive evaluation through principal component analysis

2. Keywords are listed in alphabetical order.

ABSTRACT & KEYWORDS

1. Lines 14-16: The problem statement is not clearly defined

**Response:** The author redefines the research question.

Line 14-15: It is important to investigate the impact of short-term high temperature exposure on maize growth and rhizosphere soil nutrients, in comparison to long-term high temperature treatment.

INTRODUCTION

1. Line 79-80. Using the principal component analysis method to evaluate soil physicochemical properties and crop growth indexes. The sentence is hanging. Rephrase.

2. Overall, the Introduction section is well written

**Response:** The author has revised the sentence “The soil physicochemical properties and crop growth indexes are evaluated using the principal component analysis method”.

MATERIALS AND METHODS

1. Lines 89-98: What is general classification of the soil with reference to common nomenclature e.g. WRBS (FAO), USDA

**Response:** Soil classification using Chinese soil system classification principles, 2009 edition. The test soil is loessal soil (China Soil System Classification 2009, CAS).

2. Lines 89-98: Rationale for site selection should be further strengthened. What does the Loess Plateau represent in the broader crop production systems in China and similar farming systems in the world?

**Response:** In China's crop production system, the Loess Plateau exemplifies an agricultural production model that is tailored to arid and semi-arid climate conditions. Globally, the agricultural system of the Loess Plateau serves as a model for agricultural development in arid and semi-arid regions, offering valuable insights for similar areas.

3. Lines 101-113: What informed/guided the temperature and heating periods used in the study

**Response:** The author has added in the experimental design.

The temperature and heating time used in the experiment were determined by relevant research results as well as previous experimental studies and research assumptions.

4. It’s not clearly stated in the Materials and Methods the time period (duration of the experiment) the experiment was done. In addition, it is important to provide information of the days to maturity of the maize variety used in this study

**Response:** The author has added in the Materials and Methods.

The duration of the experiment was 95 days during the whole growth period of maize.

After cooling, maize seeds (Zhengdan 958) were sown in each pot using a drilling method

During the maize growth period, which lasts 95 days, measurements were taken for crop roots, plant height, and leaf length.

5. Was the soil characterized for physicochemical and biological properties before experimentation?

**Response:** The predominant soil type in the research area is loessal soil, characterized by a pH of 8.04, SOM content of 14.25 g kg^-1^, TN content of 0.58 g kg^-1^, AP content of 19.06 mg kg^-1^, AK content of 175.75 mg kg^-1^, and a silty loam texture.

RESULTS & DISCUSSION

1. Tables 1 and 2: Were the treatments significantly different? There no statistical indices for comparison of treatment means.

**Response:** The authors present the values of these indicators in a table. On this basis, a series of tests and evaluations were made using principal component analysis, and the final conclusion was obtained by the principal component statistical analysis method, which was used to determine the significant differences between different treatments. We will also take care to ensure that data and statistical analysis results are presented more clearly in future writing to improve the readability and scientific nature of the paper. Thank you.

2. Limitations of the study and grey areas for further research should be clearly articulated in the Discussion.

• The study was conducted under controlled (pot experiment) conditions, which does not represent the real field conditions where farmers grow maize. It therefore becomes challenging to extrapolate the findings to field conditions where both biotic and abiotic conditions vary in space and time.

• Measurements were only done over one cropping cycle. The study could have been conducted over 2 or more cropping cycles to validate the results. In the absence of these repeated measurements over 2 or more seasons, the authors could consider simulation modeling to validate their findings in space and time

• Limitations of Principal Component Analysis in the context of this study

**Response:** The authors add limitations to the study at the end of the discussion.

The study was conducted under controlled pot conditions, which differ from actual field cultivation of corn, posing challenges in generalizing findings to field environments with varying biological and abiotic conditions. Additionally, measurements were limited to a single planting cycle, lacking verification across multiple cycles. Future research will involve multi-cycle studies for result validation, utilizing simulation modeling to assess results across space and time. Moreover, the principal component analysis method utilized in the study has limitations, particularly in complex biological and abiotic conditions. Subsequent studies may benefit from integrating other analytical methods for a more comprehensive understanding of the data.

3. I was wondering, what are the implications of the study findings regarding management of temperature regimes in soils and impacts on soil chemical and biological properties in maize-based cropping systems. For example, how can farmers realistically manipulate soil temperatures to influence soil chemical and biological properties and crop growth?

**Response:** The specific idea of this research involves heating the surface of the soil at high temperatures, which affects its physical and chemical properties through temperature conduction. Subsequently, corn is planted after the soil is cooled to investigate the differences in corn growth following the improvement of soil properties. This study aims to offer a significant theoretical foundation and practical value for agricultural production.

The results help to understand the effects of different temperatures on soil chemistry and biological properties, thereby providing farmers with strategies to improve soil quality. Through short soil temperature treatment, the activity of soil microorganisms can be promoted, the nutrient supply capacity of soil can be improved, the soil quality can be improved, and the soil fertility and water retention can be increased.

It can stimulate researchers' research and development and exploration of high-temperature equipment, and then promote it to agricultural use, which can save energy and increase efficiency compared with traditional temperature treatment.
