# Supplementary material for: The impact of high-temperature treatments on maize growth parameters and soil nutrients: A comprehensive evaluation through principal component analysis
Source: PLoS One. 2024 Aug 15;19(8):e0309070. doi: 10.1371/journal.pone.0309070 (PMC11326588; doi:10.1371/journal.pone.0309070)
Supplement: Attachment — Submitted filename: Review Comments_PONE-D-24-13037_R1.docx [file pone.0309070.s006.docx]

**THE COMBINED EFFECT OF TEMPERATURE ON SOIL AND MAIZE WAS EVALUATED BASED ON PRINCIPAL COMPONENT ANALYSIS- PONE-D-24-13037**

This study investigated the impact of temperature-modified soil on nutrient activation and crop growth, using loess soil with low fertility and maize as the test plant. Overall, the paper was well written, with clear objectives, detailed methodology, and systematic description of the results.

**Overall Decision: Accept**

**ABSTRACT & KEYWORDS**

1. The problem statement is not clearly defined

**RESULTS & DISCUSSION**

1. I was wondering, what are the implications of the study findings regarding management of temperature regimes in soils and impacts on soil chemical and biological properties in maize-based cropping systems. For example, how can farmers realistically manipulate soil temperatures to influence soil chemical and biological properties and crop growth?

The authors gave a satisfactory response under “responses to reviewers’ comments” document but did not include the explanation in the actual Discussion section. Add the response to the Discussion section.
