# Supplementary material for: The impact of high-temperature treatments on maize growth parameters and soil nutrients: A comprehensive evaluation through principal component analysis
Source: PLoS One. 2024 Aug 15;19(8):e0309070. doi: 10.1371/journal.pone.0309070 (PMC11326588; doi:10.1371/journal.pone.0309070)
Supplement: Attachment — Submitted filename: Response to Reviewers.docx [file pone.0309070.s007.docx]

PONE-D-24-13037R1

The impact of high-temperature treatments on maize growth parameters and soil nutrients: A comprehensive evaluation through principal component analysis

PLOS ONE

Reviewer #1: It seems authors couldn’t understand point raised like on soil texure, authors explain it was change in soil particles. But texture is different soil property. Authors once again requested to go the the comments carefully and give proper reasoning. This must also be reflected in revised MS. Superficial reply of the issues raised must be avoided.

Response: I am very sorry that the author misunderstood the definition of soil texture. The author fully accepts the reviewer's definition of soil texture, which is different soil properties.

The author reanswers the question of how the texture of the soil is affected. Add in the second paragraph of the discussion. The specific contents are as follows:

It can be seen that short-term high-temperature heating significantly impacts the physical, chemical, and biological properties of soil by decreasing soil pH, reducing soil organic matter and total nitrogen content, increasing microbial activity, and enhancing the availability of potassium in the soil. Furthermore, soil properties are influenced by both soil moisture and soil type. The soil moisture can impact the rate of heat conduction and organic matter decomposition, while various types of soil exhibit distinct responses to heat due to differences in mineral composition and structure. As a result, these factors collaborate to alter the characteristics of the soil.

Reviewer #2: This study investigated the impact of temperature-modified soil on nutrient activation and crop growth, using loess soil with low fertility and maize as the test plant. Overall, the paper was well written, with clear objectives, detailed methodology, and systematic description of the results.

Overall Decision: Accept

ABSTRACT & KEYWORDS

1. The problem statement is not clearly defined

Response: The author has revised it, and the details are as follows:

In contrast to prolonged exposure to high temperatures, investigating short-term high-temperature stress can provide insights into the impact of varying heat stress durations on plant development and soil nutrient dynamics, which is crucial for advancing ecological agriculture.

RESULTS & DISCUSSION

1. I was wondering, what are the implications of the study findings regarding management of temperature regimes in soils and impacts on soil chemical and biological properties in maize-based cropping systems. For example, how can farmers realistically manipulate soil temperatures to influence soil chemical and biological properties and crop growth?

The authors gave a satisfactory response under “responses to reviewers’ comments” document but did not include the explanation in the actual Discussion section. Add the response to the Discussion section.

Response: The author has added specific replies to the last paragraph of the discussion section, further enhancing the practicality of the technique. The specific contents are as follows:

The specific concept of this research involves subjecting the soil surface to high-temperature heating, which influences its physical and chemical properties through thermal conduction. Subsequently, corn is planted after the soil has been cooled down to examine the variations in corn growth resulting from enhanced soil properties. By briefly treating the soil temperature, it can promote the activity of soil microorganisms, enhance the nutrient supply capacity of the soil, improve soil quality, and increase both fertility and water retention. This can inspire researchers to explore high-temperature equipment for research and development purposes and subsequently facilitate its application in agriculture. This approach offers energy savings and improved efficiency compared to traditional temperature treatments.
